# A Rare Incidental Discovery of an Intrapulmonary Shunt in a Young Man: A Case Report

**DOI:** 10.7759/cureus.83326

**Published:** 2025-05-01

**Authors:** Varshitha T Panduranga, Mrinal J P Oble, Ammar Y Abdulfattah, Adam S Budzikowski, Samy I McFarlane, Sabu John

**Affiliations:** 1 Department of Internal Medicine, State University of New York Downstate Health Sciences University, Brooklyn, USA; 2 Department of Cardiology, State University of New York Downstate Health Sciences University, Brooklyn, USA; 3 Department of Cardiology, State University of New York Downstate Medical Center, Brooklyn, USA

**Keywords:** agitated saline contrast studies, echocardiogram with bubble study, intracardiac shunting, intrapulmonary shunt, transthoracic echocardiogram

## Abstract

An intrapulmonary shunt (IPS) occurs when blood bypasses oxygenation in the lungs, flowing directly from the right side of the heart to the left side without undergoing gas exchange. This condition is distinct from an intracardiac shunt, which involves an abnormal connection between the heart chambers or vessels, allowing atypical blood flow. In this report, we present the case of a 21-year-old man with a one-year history of persistent cough, nocturnal chest pain, nasal congestion with shortness of breath, and generalized abdominal pain. In the emergency department, his vital signs and physical examination were unremarkable. Electrocardiography (EKG) revealed sinus bradycardia with right-axis deviation and incomplete right bundle branch block pattern. Chest X-ray and routine laboratory investigations were normal. The patient was referred to cardiology for further evaluation of shortness of breath. A transthoracic echocardiogram (TTE) with a bubble study demonstrated a normal ejection fraction of 63% with no regional wall motion abnormalities. Agitated saline injected via the left antecubital vein revealed no bubbles in the left atrium during the first six cardiac cycles. However, after six cycles, a small number of bubbles appeared in the left atrium and left ventricle, indicative of an IPS. In this case report, we highlight a unique incidental finding of an IPS in a young man, emphasizing the importance of bubble study timing in distinguishing IPS from intracardiac shunts.

## Introduction

An intrapulmonary shunt (IPS) can arise from various etiologies, including pulmonary conditions (such as pneumonia, pulmonary edema, atelectasis, or acute respiratory distress syndrome (ARDS)) and vascular abnormalities like pulmonary arteriovenous malformations and hepatopulmonary syndrome [1]. An IPS occurs when blood flows from the right side of the heart to the left side without undergoing oxygenation in the lungs, bypassing gas exchange. One of the ways this can happen is through direct connections between the pulmonary arteries and veins, often in the context of pulmonary arteriovenous malformations. This differs from an intracardiac shunt, which is caused by an abnormal connection between the heart's chambers or vessels, resulting in irregular blood flow. Echocardiography with bubble study remains the most effective method for detecting these shunts. Symptoms of IPS often include dyspnea, hypoxemia resistant to oxygen therapy, cyanosis, and exercise intolerance [2]. The timing of bubble appearance during a contrast echocardiography study is crucial for differentiating between intracardiac and IPS. In cases of intracardiac shunts, bubbles typically appear in the left atrium within 1-2 cardiac cycles after their introduction. Conversely, IPS is characterized by a delayed appearance of bubbles in the left atrium, usually occurring within 4-8 cardiac cycles [3].

## Case presentation

**Figure 1:**
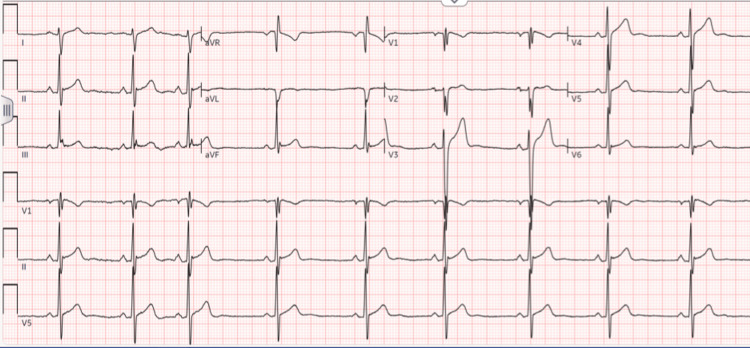
EKG showing sinus bradycardia, right-axis deviation, and incomplete right bundle branch block

**Figure 2:**
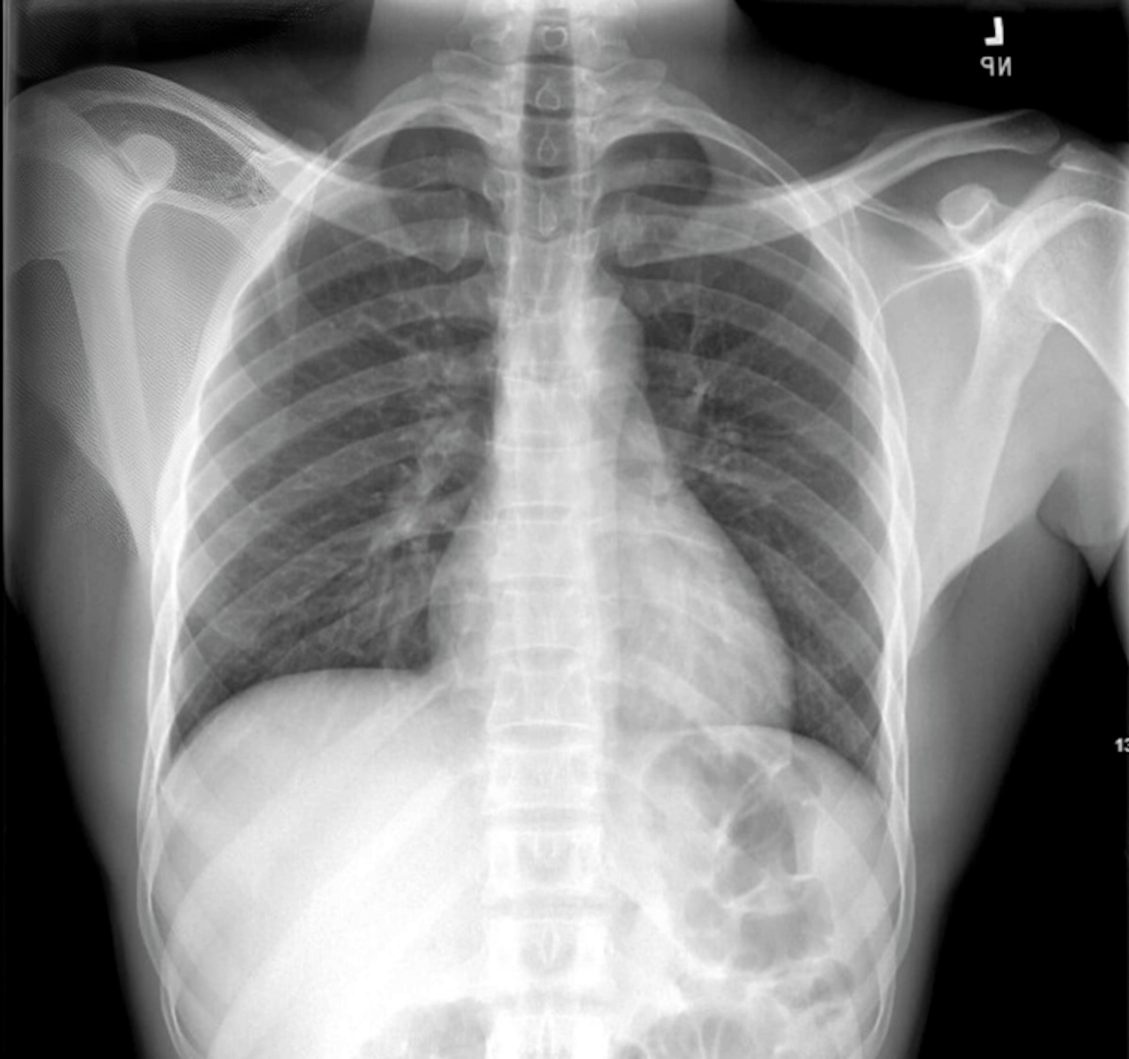
Chest X-ray showing no evidence of consolidation or atelectasis indicative of infection

A 21-year-old young man, with no significant past medical history, presented to the emergency department with a one-year history of persistent cough, nocturnal chest pain, nasal congestion with dyspnea, and generalized abdominal pain. On initial evaluation, his vital signs were stable. Physical examination revealed frontal and maxillofacial tenderness with swollen nasal turbinates, consistent with sinus congestion. The remainder of the exam was unremarkable. An electrocardiogram (EKG) showed sinus bradycardia with right-axis deviation and incomplete right bundle branch block (Figure 1), prompting further investigation. Chest X-ray (Figure 2) and routine laboratory tests (Table 1) were within normal limits. Initial differential diagnoses included chronic tuberculosis, given his prolonged cough and abdominal pain, but his interferon-gamma release assay (IGRA) for tuberculosis testing was negative. The diagnostic entities of pneumonia and pulmonary embolism were entertained but deemed unlikely, given the clinical presentation, laboratory findings, and diagnostic imaging. The patient was treated symptomatically for allergic rhinitis with intranasal fluticasone and advised to follow up with cardiology for further evaluation of shortness of breath.

**Table 1:** Laboratory findings BUN: blood urea nitrogen, ALT: alanine aminotransferase, AST: aspartate aminotransferase, eGFR: estimated glomerular filtration rate, WBC: white blood cell, RBC: red blood cell, MCV: mean corpuscular volume.

Labs	Results	Normal reference range
Sodium	141	136-146 mmol/L
Potassium	3.6	3.5-5 mmol/L
Chloride	103	98-106 mmol/L
CO_2_	29	24-31 mmol/L
BUN	10.0	6.0-20.0 mg/dL
Creatinine	0.88	0.7-1.2 mg/dL
ALT	15	0-41 U/L
AST	16	10-50 U/L
Total bilirubin	0.6	0-1.2 mg/dL
Calcium	9.8	8.6-10 mg/dL
Albumin	4.5	3.3-6.1 g/dL
Total protein	7.7	6.4-8.3 g/dL
Alkaline phosphatase	76	35-145 U/L
eGFR	>60	≥60.0 mL/min/1.73 m^2^
WBC	4.67	4.5-10.9 K/uL
RBC	5.49	4.2-6.10 M/uL
Hemoglobin	16.5	14-18 g/dL
Hematocrit	48.1	42-52%
MCV	87.6	78-95 fL
Platelet	178	130-400 K/uL
Anion gap	9	5-15 mEq/L

**Figure 3:**
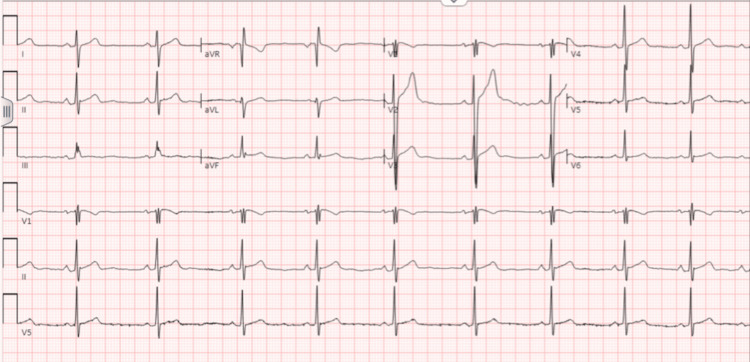
EKG showing persistent sinus bradycardia and incomplete right bundle branch block

**Figure 4:**
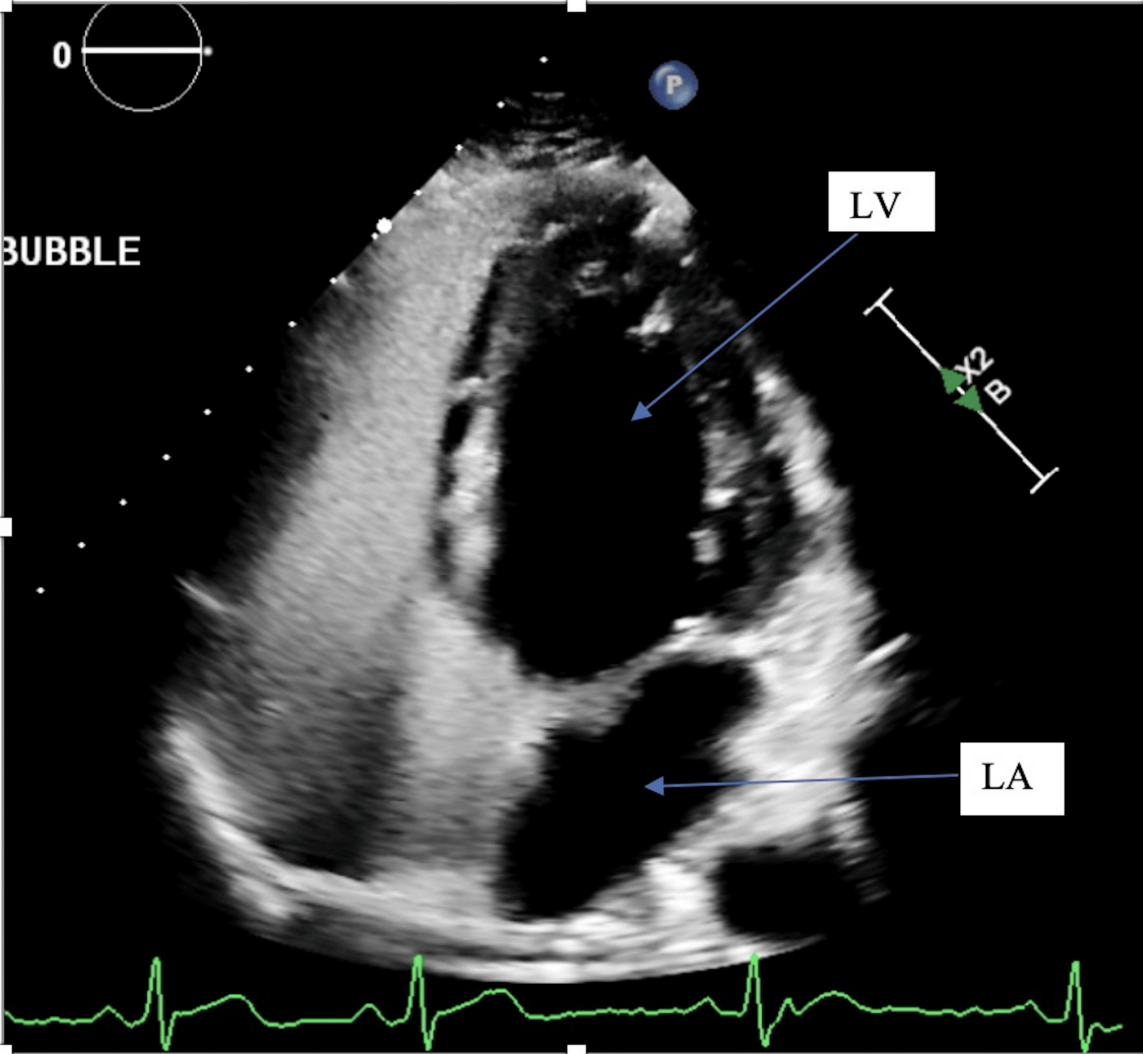
Echocardiogram showing no evidence of bubble in the left ventricle (LV) or left atrium (LA) within the six cardiac cycles

**Figure 5:**
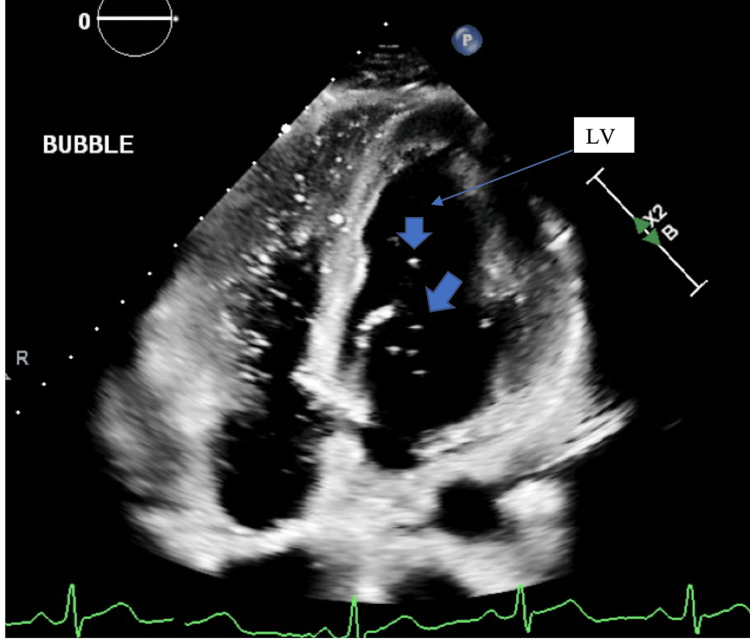
Echocardiogram showing bubbles (arrows) visible in the left ventricle (LV) after the six cardiac cycles

Two months later, the patient presented to the cardiology clinic for further evaluation. He reported that he was running out of his fluticasone prescription, resulting in the recurrence of nasal congestion, but denied new or worsening symptoms. Repeat EKG (Figure 3) showed the same findings of sinus bradycardia and incomplete right bundle branch block. Given the persistent EKG findings, an echocardiogram with a bubble study was performed (Figures 4 and 5) to assess left atrial and ventricular size, and global and segmental wall motion and to rule out an atrial septal defect (ASD). The echocardiogram revealed a normal left ventricular ejection fraction of 63% with preserved wall thickness and normal systolic and diastolic function. The left and right atria, as well as the right ventricle, were normal in size and function. No abnormalities were detected in the mitral, aortic, tricuspid, or pulmonic valves, and the interatrial and interventricular septa were intact. During the bubble study, agitated saline was injected through the left antecubital vein. No bubbles were observed crossing into the left atrium within six cardiac cycles, as seen in Figure 4, either at rest or with the Valsalva maneuver, ruling out an intracardiac shunt. However, a small number of bubbles appeared in the left atrium and left ventricle after six cardiac cycles, indicating the presence of an IPS, as seen in Figure 5.

## Discussion

Agitated saline contrast studies are a vital aspect of modern echocardiography. This technique is crucial for distinguishing between intracardiac shunt and IPS, often influencing therapeutic decisions. Its significance has grown with the development of percutaneous treatment options for atrial septal defects and patent foramen ovale (PFO) [3]. The bubble study differentiates intracardiac and IPS based on the timing of contrast appearance in the left heart, reflecting distinct physiological mechanisms. Intracardiac shunts are diagnosed by a positive bubble study within 1-2 cardiac cycles, confirmed by TTE or transesophageal echocardiogram (TEE) with color Doppler evidence of PFO or ASD, while IPS is identified by delayed contrast appearance within 4-8 cardiac cycles, with no evidence of PFO or ASD on imaging [4]. An echocardiographic bubble study involves creating contrast bubbles by forcefully mixing air, sterile saline, and the subject's blood between two syringes connected to an IV catheter placed in the antecubital or median basilic vein. The agitated solution is then injected forcefully while obtaining simultaneous images in the apical four-chamber view to detect intracardiac shunting [5,6]. Contrast echocardiography distinguishes IPS from intracardiac shunting based on the timing of bubble appearance in the left heart. In the absence of right-to-left shunting, contrast bubbles remain in the right heart and are filtered by the pulmonary circulation. Rapid left-heart opacification after right-heart filling indicates intracardiac shunting, whereas delayed opacification (≥3 cardiac cycles) suggests transpulmonary passage through distended pulmonary capillaries or arteriovenous shunts. Contrast echocardiogram with harmonic imaging enhances bubble detection, improving the accuracy of shunt localization [7,8]. Transthoracic echocardiography with agitated saline contrast injection not only is effective in identifying right-to-left atrial communications but also provides crucial insights into IPS. Its sensitivity and specificity make it superior to other techniques, reinforcing its role as an essential tool in diagnosing and guiding the management of shunting disorders [9].

Several diagnostic modalities, beyond the commonly used echocardiographic bubble study, are available for identifying IPS, each offering unique strengths and applications depending on the clinical context. Lung ultrasound is another valuable bedside tool for diagnosing IPS by identifying a tissue-like pattern indicating lung consolidation and using color Doppler to detect well-perfused vessels within non-aerated lung segments [10]. However, the sensitivity and specificity of this diagnostic modality are not well established. Another mode of diagnosis, the technetium-99-labelled macroaggregated albumin (MAA) scan, is a reliable method for diagnosing IPS by identifying right-to-left shunting through the detection of radiotracer uptake outside the lungs, such as in the brain or kidneys. The bubble study is more sensitive for detecting smaller shunts, while MAA provides more precise quantification but may miss very small shunts [11]. Other modalities of diagnosing IPS include ventilation-perfusion scanning, computed tomography pulmonary angiography, arterial blood gas analysis, and pulse oximetry with oxygen challenge test, to name a few. However, bubble studies are particularly effective for real-time, qualitative assessment of shunts, especially in acute care settings. 

The presence of clinically significant IPS, where blood bypasses alveolar-capillary gas exchange units, is a hallmark of various cardiac and noncardiac diseases, often resulting from abnormal blood flow through malformed vascular structures [12]. Intrapulmonary arteriovenous shunting in humans is primarily caused by dilated, pre-capillary arteriovenous communications within the lung parenchyma, referred to as supernumerary vessels, which branch off pulmonary arteries and connect directly to pulmonary veins [13]. These vessels potentially contribute to inducible pulmonary arteriovenous shunting by diverting blood away from alveolar capillary networks [14]. Echocardiographic evidence of pulmonary arteriovenous shunting beyond the neonatal period is rare and, when observed, is often associated with extrapulmonary liver disease. However, the patient in this case report, despite showing no signs of liver disease, exhibited symptoms likely caused by IPS, making this a unique presentation.

IPS presents with non-specific symptoms like dyspnea, cyanosis, fatigue, chest discomfort, and tachypnea, with severity varying based on the underlying cause. For example, hepatopulmonary syndrome may present with jaundice and ascites, while pneumonia-related shunting can include fever and pleuritic chest pain. Symptoms are often non-specific, making diagnosis challenging and requiring ruling out other conditions. If untreated, IPS can lead to severe hypoxemia, respiratory failure, and even mortality. Diagnosis involves imaging techniques such as bubble studies, lung ultrasound, and MAA scans, while management focuses on treating the underlying condition and supporting oxygenation. Prognosis depends on the cause, with potential for improved outcomes with proper intervention but risk of chronic hypoxia and complications in severe cases.

## Conclusions

This case highlights the incidental finding of IPS in a young adult with chronic symptoms, where an echocardiographic bubble study played a pivotal role in the diagnosis. It emphasizes the importance of considering intrapulmonary shunts in the differential diagnosis, even when the underlying cause is unclear, and underscores the value of bubble studies in guiding clinical decisions and improving patient outcomes in atypical presentations.
